# Beyond Mortality: Severely Frail Femur Fracture Patients Can Regain Independence after Surgery

**DOI:** 10.3390/jcm13113197

**Published:** 2024-05-29

**Authors:** Noa H. M. Ponds, Jochem H. Raats, Devon T. Brameier, Henk Jan Schuijt, Lisa Cooper, Abigail Sagona, Houman Javedan, Michael J. Weaver

**Affiliations:** 1 Department of Orthopedic Surgery, Brigham and Women’s Hospital, Harvard Medical School, Boston, MA 02215, USA; 2 Department of Surgery, St. Antonius Hospital, 3543 AZ Utrecht, The Netherlands; 3 Center for Geriatric Trauma, St. Antonius Hospital, 3543 AZ Utrecht, The Netherlands; 4 Division of Aging, Department of Medicine, Brigham and Women’s Hospital, Harvard Medical School, Boston, MA 02215, USA; 5 Department of Geriatric Medicine, Rabin Medical Center, Tel Aviv 4941492, Israel

**Keywords:** geriatric trauma, femur fracture, frailty, self-reported outcome

## Abstract

**Objectives:** Little is known about the post-operative functional outcomes of severely frail femur fracture patients, with previous studies focusing on complications and mortality. This study investigated patient- or proxy-reported outcomes after femur fracture surgery in older adult patients with severe frailty. **Methods:** This was a retrospective cross-sectional study of older adult (>70 years) patients with severe frailty (defined by a Comprehensive Geriatric Assessment-based Frailty Index (FI-CGA) ≥ 0.40), who underwent femur fracture surgery at a Level 1 Trauma Center. Patients or their proxy (i.e., close relative) reported mobility, psychosocial, and functional outcomes at least 1-year after surgery. **Results:** Thirty-seven predominantly female (76%) patients with a median age of 85 years (IQR 79–92), and a median FI-CGA of 0.48 (IQR 0.43–0.54) were included. Eleven patients (30%) regained pre-fracture levels of ambulation, with twenty-six patients (70%) able to walk with or without assistance. The majority of patients (76%) were able to have meaningful conversations. Of the patients, 54% of them experienced no to minimal pain, while 8% still experienced a lot of pain. Functional independence varied, as follows: five patients (14%) could bathe themselves; nine patients (25%) could dress themselves; fourteen patients (39%) could toilet independently; and seventeen patients (47%) transferred out of a (wheel)chair independently. **Conclusions:** Despite the high risk of mortality and perioperative complications, many of the most severely frail patients with surgically treated femur fractures regain the ability to ambulate and live with a moderate degree of independence. This information can help healthcare providers to better inform these patients and their families of the role of surgical treatment during goals of care discussions.

## 1. Introduction

Increasing life expectancy and advancements in healthcare has resulted in a rise in the number of older adult patients presenting with a hip or femur fracture, as well as an increase in the degree of frailty these patients are living with [1,2]. Frailty is a dynamic biopsychosocial syndrome characterized by a decreased reserve to regain homeostasis after a stressor event. This is due to a pooled decline of multiple domains (including among them genetics, biology, function, cognition, nutrition, psychosocial well-being, and socio-economic status), leading to an inability to recover to baseline and, thus, a greater vulnerability and decreased resilience to adverse events such as falls, fractures, institutionalization, and death [3,4,5,6,7,8]. Among patients who suffer hip fractures, frailty has been associated with a higher risk of perioperative complications and mortality [9,10,11]. As a result, these frail patients require greater attention from both orthopedic surgeons and geriatricians, demanding multiple medical consultations to manage their many comorbidities. These patients often have extended lengths of stay (LOS), delayed surgery, and ultimately increased mortality [12,13,14,15]. Patients with severe frailty are entering a slow decline and approaching the end of their life. When managing femur fractures in these patients, it becomes increasingly important to balance expectations and benefits of surgery with the possible harm of surgical treatment, aligning patients’ goals of care with expected outcomes.

Multidisciplinary management and treatment approaches, such as Orthogeriatric Co-management (OGC), have been shown to improve the outcomes and reduce the economic healthcare burden of geriatric fractures [11,16]. Frailty indices have been developed in an attempt to better capture patients’ overall medical condition by accounting for age, medical comorbidities, and their functional and cognitive status. One example is the Comprehensive Geriatric Assessment (CGA)-based Frailty Index (FI-CGA), which assesses frailty in older adults by accounting for the accumulation of deficits and reserves based on variables collected during the CGA [17,18]. This includes (but is not limited to) patient-reported variables such as mood, changes in memory, polypharmacy, comorbidities, and functional variables [19]. The FI-CGA better predicts outcomes based on patients’ physiological age as opposed to chronological age, with higher FI-CGA scores reflecting a higher risk for adverse outcomes and mortality [20,21,22,23,24].

There is a paucity of literature surrounding the long-term patient-reported outcomes of severely frail patients undergoing femur fracture surgery. Prior studies have focused on post-operative complications and mortality in these patients [25,26,27]. As this population often faces cognitive impairment, end-of-life care, and a continuous functional decline regardless of their injury, collecting appropriate functional measures is challenging. This difficulty has limited the available literature on this population, leading to these patients being assumed to face inevitable short-term mortality and poor patient outcomes following femur fractures [28]. In order to improve shared decision-making, it is important to understand the expected patient-centered outcomes of this specific population to inform goals of care discussions, and to align the expectations of patients and their families.

The objective of this study is to evaluate the functional outcomes of severely frail patients with femur fractures in terms of ambulatory status, pain relief, and activities of daily living.

## 2. Materials and Methods

This is a single-center, retrospective, cross-sectional study assessing the long-term functional outcomes of all consecutive severely frail older adult patients with surgically treated femur fractures (AO 31–33 A-C, including periprosthetic femur fractures) [29,30] over a 3-year period.

Patients were identified from electronic patient databases using the International Classification of Diseases Clinical Modification (ICD-CM) fracture codes (S72.0–S72.9) [31] and a review of their Electronic Health Record (EHR). Inclusion criteria were severely frail geriatric patients (as determined by having a FI-CGA ≥ 0.4 and an age of 70 years or older) with a surgically managed femur fracture, at least 1-year of follow-up, and a completed CGA by a geriatrician prior to their surgery with a calculated FI-CGA.

Eligible patients or proxies of patients were called and surveyed about patient-reported outcomes at what they considered the best point in the patient’s recovery after surgery, in terms of functional ability. This patient-specific “best point in their recovery” was utilized as the time-point for measuring peak functional outcomes because the goal was to capture the maximal functional benefit gained from surgery in a population of patients who are in a constant physiological decline. As the rate of decline may vary from patient-to-patient, a patient-specific time-point for the “best point in their recovery” was selected as more appropriate for the outcomes of interest than an arbitrary single time-point in the recovery period. If patients were discharged to an institution and the patient or proxy could not be reached directly, the institution was called to make an attempt of contact with the patient or proxy. Patients were excluded if there was no response from the patient and/or proxy after a maximum of 3 attempts of contact, or if follow-up data (i.e., deceased and/or no available proxy, or no available information from the discharge institution) were not available.

In the study institution, a CGA is performed by geriatricians as part of the standard of care for all femur fracture patients aged 70 and above. The CGA is a multi-domain assessment of aging which includes cognitive, functional, and social assessments, in addition to a medication review and physical examination [19]. Information on all healthcare domains included in the calculation of the frailty index are collected within the CGA, which is then used to calculate an index score (FI-CGA) based on a deficit accumulation model of frailty, by using an integrated algorithm within the EHR as described elsewhere [17,18]. Frailty is defined as having a FI-CGA of 0.2 or higher. It has been shown that a FI-CGA ≥ 0.41 is associated with more cognitive and functional impairment. Greater frailty also showed a clear dose-dependent relationship (more = worse) with respect to community and ambulatory service use [18]. Moreover, a FI-CGA > 0.45 is related to higher mortality in community-dwelling adults [24].

In this study, a FI-CGA ≥ 0.4 was used to define severe frailty. A typical patient with an FI of 0.4 would be cognitively impaired, dependent on aids and/or others in terms of mobility and ADLs, and have a limited life expectancy. Most patients either live in an assisted living environment, or are close to needing that level of care. That being said, the severely frail patient population is very heterogenous in characteristics and outcomes, as dependency can manifest in all different domains of life.

Patient- or proxy-reported outcomes were chosen to reflect the absolute minimum requirements for a life with acceptable quality. Subjects were asked to recall the best point of the patients’ recovery in terms of mobility, psychosocial, and functional outcomes. Mobility outcomes were assessed by ambulatory status (i.e., independent, with a walking aid, with assistance from others, not able to walk, or wheelchair-bound at all times), and whether patients recovered to their pre-fracture ambulation level. Psychosocial outcomes included post-operative living situation (i.e., living independently at home, living at home with assistance for activities of daily living, living with family or friends, or living in an institutional care facility), whether patients were able to have a meaningful conversation with others, and a 4-point Likert scale for pain experienced at the peak of recovery. Functional outcomes were assessed using the Katz ADL questionnaire for activities of daily living (ADL) [32,33]. Patients were asked about their level of independence (i.e., fully independent, help required from others or completely dependent) in the following categories: bathing, dressing, toileting, transferring in and out of a chair or wheelchair. Additionally, patients were asked if they were able to control their bladder and/or bowel movements, and if they were able to feed themselves (i.e., without assistance, help required from others, unable to feed themselves, or required tube feeding or parental nutrition). Missing values were excluded from analyses.

Descriptive statistics were used to report quantitative variables. Continuous variables were reported with median and interquartile ranges, and categorical variables with numbers and percentages. This was a purely descriptive study, and no comparison was made between groups. All statistical analyses were conducted using IBM SPSS Statistics™ for Windows, Version 28.0 (IBM Corp., 2021, Armonk, NY, USA). Data collection was carried out using Research Electronic Data Capture (REDCAP) version 14.0.27, an online Health Insurance Portability and Accountability Act (HIPAA)-compliant data management tool [34].

This study was registered with the Institutional Review Board (2019P003694), and was performed in accordance with the ethical standards laid down in the 1964 Declaration of Helsinki and its later amendments. This article was written in accordance with the Strengthening the Reporting of Observational Studies in Epidemiology (STROBE) guidelines for cross-sectional studies of the EQUATOR™ network [35].

## 3. Results

**Figure 1:**
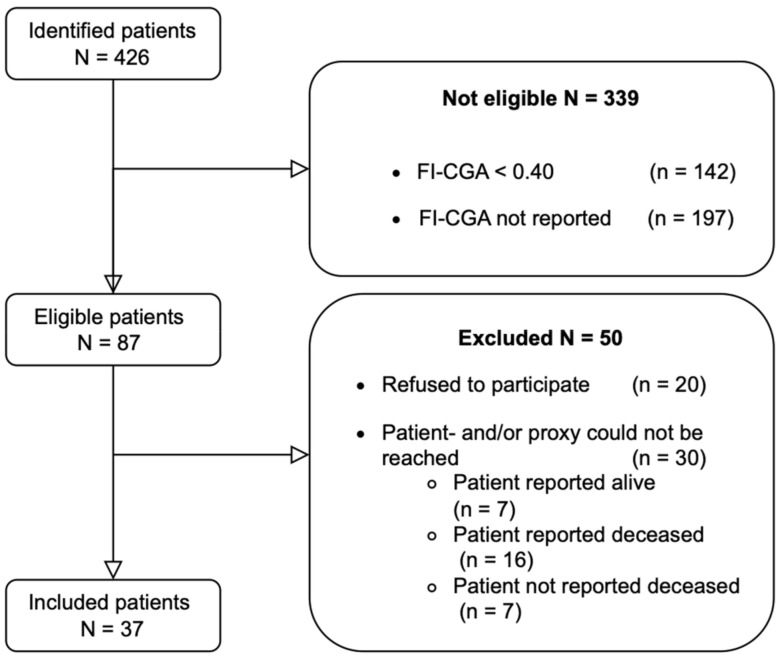
CONSORT Flow diagram of patient screening and inclusion.

From a total of 426 patients treated for femur fractures, 87 were eligible for this study (Figure 1). After contacting eligible patients and/or their proxies to complete the survey, 37 patients participated and were included for analysis (42% response rate). Nine patients (24%) had died at the time of contact, so their proxies were asked to complete the survey in their stead. Reasons for eligible patients not participating in the study included not being willing to participate in the study or neither the patient nor proxy being able to be reached to complete the survey. Of all eligible patients, at least 25 patients had died at the start of the study, resulting in an overall mortality rate of 29%. The final cohort consisted of the 37 patients with outcomes collected either directly from the patient or their proxy.

*Patient Demographics:* At the time of injury, the median age was 85 years (IQR 78–92), with 70% of patients aged ≥80 (Table 1). Patients had a median FI-CGA of 0.48 (IQR 0.43–0.54) and were predominantly female (n = 28, 76%). Thirty-four patients were classified as high risk or very high risk, based upon the American Society of Anesthesiologists (ASA) score (ASA class ≥ III). Most patients (n = 29, 78%) presented with a proximal femur fracture, followed by six patients (16%) with a distal femur fracture, and two patients (5%) with a femoral shaft fracture.

**Figure 2:**
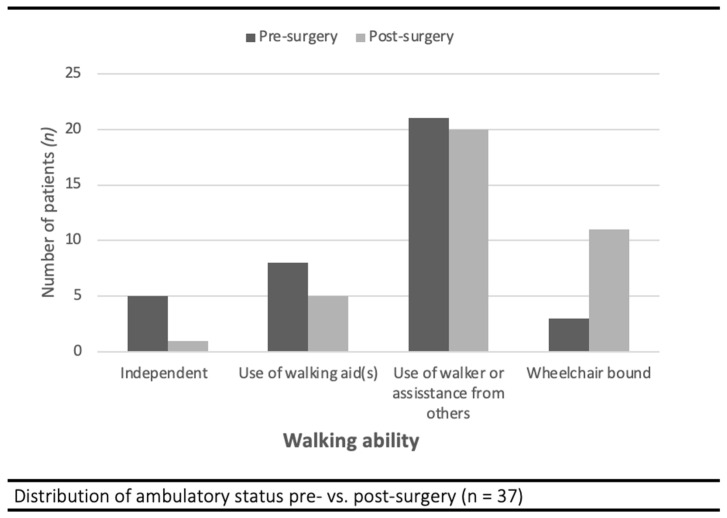
Ambulatory status of severely frail patients before and after femur fracture fixation.

*Physical Function:* Prior to injury, five patients (14%) were able to walk independently, twenty-nine patients (78%) could walk with walking aid(s) or a walker, and three patients (8%) were wheelchair-bound. At the best point in their recovery, one patient (3%) was able to walk independently, and twenty-five patients (69%) used walking aid(s) or a walker. Eleven patients (30%) were not able to walk at all or were wheelchair-bound, of whom five made use of a walker and three were already wheelchair-bound before injury. A total of eleven patients (30%) had recovered to the level of ambulation they had prior to their fracture (Figure 2). In terms of pain, twenty (54%) patients reported no to minimal pain, twelve patients (33%) still experienced mild to moderate pain, and three (8%) patients still experienced severe pain, although it is unclear if this pain related to their femur or other medical problems.

**Figure 3:**
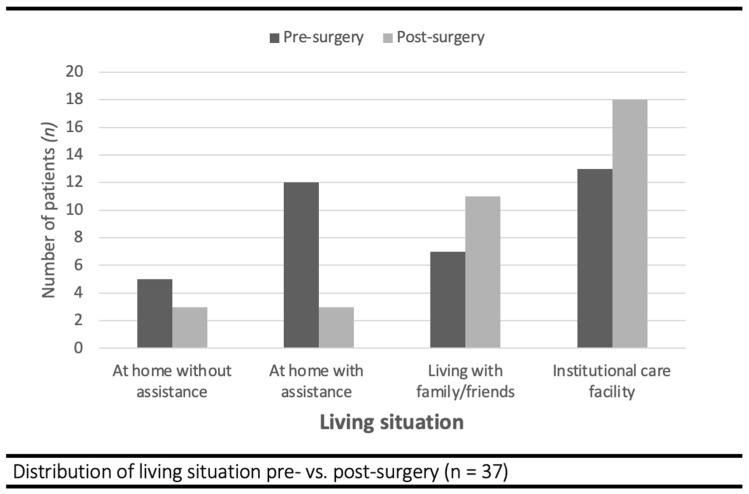
Living situation of severely frail patients before and after femur fracture fixation.

*Psychosocial Outcomes:* Prior to their femur fracture, seventeen patients (45%) lived at home, with or without assistance for ADL, seven (19%) patients lived with family or friends, and thirteen patients (35%) lived in an institutional care facility (Figure 3). At the best point in their recovery, six patients (16%) lived at home independently or with assistance for ADL, eleven patients (30%) lived with family or friends, and eighteen patients (49%) lived in an institutional care facility. Most patients (76%) reported that they were able to have a meaningful conversation with others.

*Activities of Daily Living:* At the best point in their recovery, five (14%) patients were able to bathe themselves completely independent, twenty-five (69%) needed assistance with bathing, and six (17%) could only be washed in bed (Table 2). A total of thirty-one (84%) could dress themselves with or without assistance, and five (14%) were unable to dress themselves. Fourteen (39%) patients required no help from others when toileting, seventeen (47%) were able to go to the toilet with assistance from others, and five (14%) were unable to go to the toilet. Seventeen (47%) patients were able to transfer out of a (wheel)chair without assistance, while thirteen (36%) required help from others, and six (17%) were unable to transfer. Most patients were able to control their bladder and bowel movements (60%), and could eat without any assistance (78%), though five (14%) patients required tube feeding or parenteral nutrition.

## 4. Discussion

Femur fractures, particularly in the severely frail older adult population, represent a sentinel event in the decline towards the end of life, associated with a high degree of morbidity and mortality [3,4,5,9,10,11]. The prior literature has focused on post-operative complications and mortality, neglecting the long-term functional outcomes of these patients [25,26,27]. This cohort of 37 femoral fracture patients living with severe frailty, which mirrors Jones et al.’s distribution of frailty [18] and prior reported OGC care teams’ target populations, demonstrates that while there is a high rate of 1-year mortality following surgical treatment of femur fractures, at the best point of recovery patients report overall positive outcomes in terms of freedom from pain, ambulatory ability, functional independence, and social interaction [5,11,27,36,37].

In terms of mobility, 30% of patients recovered to their previous ambulatory level, and 69% of patients were able to mobilize using a walking aid or walker in the 1-year following their injury. Functional independence varied per task and level of difficulty, with only 13% of patients able to bathe themselves independently, while almost 50% of patients were able to transfer from a chair. Various studies have reported on the loss of function and mobility in geriatric trauma patients [10,38,39,40], but none specifically captured the severely frail femur fracture population. For example, Magaziner et al. [38] reported that, among 536 hip fracture patients over 65 years older, nearly two-thirds of patients regained their pre-fracture walking ability, a little less than half of them had recovered physical activities of daily living, and just under one-third had a recovered IADL performance level. A Dutch prospective cohort study investigating the long-term ambulatory ability of previously ambulatory hip fracture patients aged above 65 years reported that 40% of patients remained ambulatory, but increasingly relied on assistive devices. Additionally, 12% of previous community ambulators transitioned to household ambulators, and 8% became non-functional ambulators [39]. Cooper et al. reported that, after 1-year, 60% of hip fracture patients lost independence in at least one essential activity of daily living (e.g., bathing or dressing themselves) [40]. These studies found significant loss of function and independence among older adults with femur fractures; however, they did not consider the impact of frailty on these outcomes, thus limiting the ability of clinicians to accurately predict severely frail patients’ return to ambulation. The current study demonstrates that, even in the setting of severe frailty, the vast majority of patients report good freedom from pain and regain some ambulatory ability, even though for most there was a decline from their pre-injury level.

Psychosocially, over three-quarters of the studied patients were able to have a meaningful conversation, and the majority had minimal pain at the best point of their recovery. Before their femur fracture, seventeen patients lived at home compared to six patients 1-year post-injury, indicating that eleven patients (30%) were institutionalized or needed to move in with family or friends after their injury. This finding is higher than the proportion of 10–20% reported in the general hip fracture population within Western industrialized countries [9], but in line with a smaller Dutch study showing that just over half of surviving patients returned to their original living circumstances 4-months following admission for a hip fracture [10]. In the severely frail patient population, sustaining a femur fracture is a life changing event, often leading to institutionalization and loss of independence, to some extent.

This study has several limitations. Given the target population, there was an expectedly low response rate of 42%, introducing a risk for non-response bias; however, the low response rate seen mirrors previously conducted patient- and/or proxy-reported survey studies among fracture patients, with response rates ranging between 38 and 66% [38,39,41,42,43,44]. Moreover, the response rate was impacted by the number of patients who had already died by the time of survey administration. We attempted to mitigate this limitation by including proxy-reported outcomes for patients who had died if proxies were reachable. The overall mortality rate of 29% among eligible patients highlights the substantial impact of femur fractures on the older frail trauma patient, and the challenges of studying long-term outcomes in this specific population. The presents study’s subjective character and the reliance on retrospective observations of patients and proxies introduces the potential for recall bias. However, considering the current pitfalls in the field of geriatric trauma, where older frail patients are prone to selective loss to follow-up, selection bias, and survival bias [45], effort should be made to include these patients in clinical studies, including by proxy representation, as these patients might benefit most from the study’s findings. Further, these patients are often on a declining medical trajectory, and it is difficult to ascribe their mortality and functional problems to their injury or surgery. Despite these limitations, this study is an important addition to the literature, and thus useful for clinicians with respect to shared decision-making, as it is the first and only study to examine and report on long-term patient-centered outcomes in severely frail femur fracture patients. To further validate current findings, future research should focus on extending follow-up to at least 2-years to better understand the long-term functional outcomes in this population. Moreover, this study made it evident that severely frail patients do achieve favorable functional outcomes after surgery, even with high mortality rates; this is, in a way, “hypothesis-forming”. Future research studies are needed to explore the factors that contribute to poor and good recovery following femur fracture surgery, as well as appropriate treatment and intervention algorithms approaching these injuries in the frail population. Additionally, a previous investigation by our research team found a good correlation between patient- and proxy-reported pain interference and physical function in ambulatory, cognitively intact older patients [43], but it remains unknown how proxies’ answers correlate with patients’ psychosocial and functional outcomes. To better understand the use and value of proxy-reported measures, future research should investigate this relationship across other domains that apply to older adult and frail orthopedic trauma patients.

The primary focus of this study was to go ‘beyond mortality’, and it is the first study to gain insight in long-term functional and psychosocial outcomes of severely frail femur fracture patients. While femur fractures clearly represent a sentinel event for older adults, as both injury and its treatment are associated with a high risk of mortality, it is evident that a considerable share of patients regain the ability to ambulate with assistance, and report good pain relief. Despite some decline in their ability to live fully independently, many severely frail femur fracture patients continue to live with a certain degree of independence. This information can help treating physicians and surgeons to better counsel patients and their loved ones during shared decision-making conversations by informing them of what to expect functionally following surgery for femur fractures in the severely frail population. In this patient-centered approach, alignment of treatment and goals of care may improve quality of life and patient/proxy satisfaction with care. Further research is needed to investigate the factors that impact patient- and proxy-reported outcomes in this patient population, as well as the additional recourses needed to care for this patient population, to facilitate better alignment of treatment decisions and patient goals of care.

## Figures and Tables

**Table 1:** Patient characteristics (n = 37).

Patient Characteristics		N = 37 *
Age		85 (78–92)
Age ≥ 80		26 (70%)
Female		28 (76%)
ASA status	I	0 (0.0%)
	II	3 (8%)
	III	24 (65%)
	IV	10 (27%)
Frailty Index		0.48 (0.43–0.54)
Surgical information		
Type of fracture		
Proximal femur		29 (78%)
- Neck of femur		9
- Intertrochanteric		17
- Periprosthetic total hip		3
Shaft of femur		2 (5%)
Distal femur		6 (16%)
- Distal femur		5
- Periprosthetic total knee		1
Type of surgery		
Hip hemiarthroplasty		6 (16%)
Closed reduction and percutaneous pinning		1 (3%)
Intramedullary nail		16 (44%)
Dynamic hip screw		5 (14%)
Femur plating		5 (14%)
Revision total joint arthroplasty and/or femur plating		4 (8%)
Mortality		
Deceased at time of contact		9 (24%)

* Values are presented as median (inter quartile range) or number (n (%)).

**Table 2:** Activities of Daily Living (ADL) of severely frail patients at the best point in their recovery following femur fracture fixation.

ADL Domains	N = 36 * (%)
Bathing	
Without assistance	5 (14)
With assistance from others	25 (69)
Sponge bath required	6 (17)
Dressing	
Without assistance	9 (25)
With assistance from others	22 (61)
Completely dependent	5 (14)
Toileting	
Without assistance	14 (39)
With assistance from others	17 (47)
Unable to go/use of urine bag	5 (14)
Transferring	
Without assistance	17 (47)
With assistance from others	13 (36)
Completely dependent	6 (17)
Bladder and/or bowel control	
In control	22 (61)
No control	14 (39)
Feeding	
Without assistance	28 (78)
With assistance from others	3 (8)
Completely dependent/tube- or parental nutrition	5 (14)

* Values are presented as number (n (%)); outcomes of ADL domains were missing for one patient.

## Data Availability

The data presented in this study are available on request from the corresponding authors. The data are not publicly available due to legal restrictions.
